# RECORD: Reference-Assisted Genome Assembly for Closely Related Genomes

**DOI:** 10.1155/2015/563482

**Published:** 2015-10-19

**Authors:** Krisztian Buza, Bartek Wilczynski, Norbert Dojer

**Affiliations:** Faculty of Mathematics, Informatics and Mechanics (MIM), University of Warsaw, Banacha 2, 02-097 Warsaw, Poland

## Abstract

*Background*. Next-generation sequencing technologies are now producing multiple times the genome size in total reads from a single experiment. This is enough information to reconstruct at least some of the differences between the individual genome studied in the experiment and the reference genome of the species. However, in most typical protocols, this information is disregarded and the reference genome is used. *Results*. We provide a new approach that allows researchers to reconstruct genomes very closely related to the reference genome (e.g., mutants of the same species) directly from the reads used in the experiment. Our approach applies de novo assembly software to experimental reads and so-called pseudoreads and uses the resulting contigs to generate a modified reference sequence. In this way, it can very quickly, and at no additional sequencing cost, generate new, modified reference sequence that is closer to the actual sequenced genome and has a full coverage. In this paper, we describe our approach and test its implementation called RECORD. We evaluate RECORD on both simulated and real data. We made our software publicly available on sourceforge. *Conclusion*. Our tests show that on closely related sequences RECORD outperforms more general assisted-assembly software.

## 1. Background

The emergence of population genomic projects leads to an ever growing need for software and methods that facilitate studying closely related organism with next-generation sequencing technologies. This includes determination of the genomic sequences of individuals in the presence of the more generic reference genome of the species. This task is known as reference-assisted genome assembly and many ongoing research projects depend on the accurate solution for this problem.

In recent years, next-generation sequencing technologies have brought us the possibility to simultanously sequence millions of short DNA fragments in a DNA library prepared from almost any biochemical experiment [1]. Great improvement in the quality and amount of short reads obtained from a single experiment allowed for development of many more biochemical assays [2] such as MNase-seq [3], DNAse-seq [4], or Chia-Pet [5] in addition to the more standard ChIP-Seq [6] or RNA-seq [7]. Similarly, the next-generation sequencing techniques may be applied to metagenomic samples returning short reads originating from multiple genomes including some potentially unknown species.

Importantly, many of these techniques require the prior knowledge of the reference genome of the species for which the experiment was performed. This genome sequence is used to map the reads and obtain the final readout of the experiment as the read counts per base pair. Such procedures are guaranteed to work very well only under the assumption that we know the exact sequence of the genome under study. There are, however, many biologically relevant cases when this assumption cannot be satisfied. For example, in quickly growing cell populations such as cancer cell-lines or microbial colonies, even rare mutations can get fixed in the population very quickly. This leads to situations where sampled sequences can significantly differ from the original reference genome. Similarly, many lab experiments involve genetically modified cells or organisms. While these modifications are usually controlled as much as possible, the researchers frequently do not know the exact landing site of the introduced sequence or the number of copies in which it was integrated into the host genome. This has naturally serious implications for the accuracy of the results because any difference between the reference genome and the sampled one will lead to differences in the expected number of reads mappable to the reference genome at the differing position. This in turn can interfere with the measurement of the real abundance of this DNA region in the sample.

This problem can be, at least theoretically, alleviated by introducing an additional step into the process: instead of directly mapping the reads to the reference genome, we can create a “modified” assembly of the genome based on the reads from the sample and the reference genome. Then, we can use this assembly to map the reads and measure their abundance. This approach can be broken down into two major steps:

- Assembling a genome of the sampled population based on the obtained reads and the reference genome.
- Assessing the abundance of reads in genomic regions using such an improved reference sequence.

In the early years of next-generation sequencing, the first step of such an approach was impractical, as the number of reads used for an experiment like ChIP-seq was far too low and their quality was not high enough to attempt assembly of a better reference genome than the one deposited in the databases by the relevant genome consortium. However, now it is commonplace that the total of sequencing reads generated for a single experiment such as ChIA-PET might be covering the genome multiple times and, at least in case of the model organisms such as* D. melanogaster* or* C. elegans*, the read lengths might be large enough to attempt an assembly.

This approach also has another limitation. If the reference sequence is very different from the one used in the experiment, it contributes more to a problem than to a solution. Any attempt to use a completely unrelated sequence as a reference in such an approach is bound to introduce errors. Therefore, in order to ensure that the output of the assembly is useful, when we provide a method of generating reference-assisted assemblies, it is crucial to validate that the reference is actually close enough to the target genome.

In this paper, we focus on developing an approach for reference-assisted genome assembly. We assume that the actual genome of the organism and the reference genome are close to each other; for example, the reference genome of the species under consideration is given, but not the genome of the particular mutant. We point out that currently used straightforward solutions produce suboptimal or, in some cases, even misleading results. For example, when simply assembling the genome from the given reads, due to the low coverage of those reads, we may obtain too short contigs leading to an assembly useless in practical applications. Consequently, we need an assembly technique which fulfills the following criteria. First of all, it should output sequences that are long enough even in cases when the coverage of the genome sequence by the experimental reads is relatively low. Second, not only should the output sequences be large enough individually, but, together, they should cover as much as possible of the genome in order to allow detection of the abundance of reads in any region of the genome. Third, the result of the assembly should be accurate; that is, the assembled genome should be as close as possible to the actual genome of the studied organism. Last, but not least, we aim to provide a* simple* assembly approach. With simplicity, we mean computational time (in order to keep the entire process computationally tractable) and the method clarity needed for ease of reproducibility and reuse of presented ideas in the context of different specific protocols. We believe the adaptation of the ideas presented in this paper may be straightforward in some applications, including RNA-Seq and ChIP-Seq. In other cases, it would be possible after substantial effort. For example, metagenomic sequencing might potentially benefit from some ideas presented in this paper. However, the currently presented approach would need to accommodate multiple genomes and reads originating from different, related species that may be present in the sample at the same time.

The growing interest in genome assembly is also reflected by recent publications. For example, Peng and Smith [8] studied genome assembly from the theoretical point of view and showed that various combinatorial problems related to genome assembly are NP-hard. On the other hand, various methods have been proposed for reference-assisted genome assembly, such as Amos [9], RACA [10], ARACHNE [11, 12], IMR/DENOM [13], RAGOUT [14], AlignGraph [15], and the pipeline developed by Gnerre et al. [16] which was developed inside the framework provided by ARACHNE. Similarly to our approach, Gnerre et al. used a de novo assembler as a component. They mapped reads to several reference genomes and used the resulting mapping information to improve the output of the de novo assembly in subsequent steps. In contrast to Gnerre et al., we only use one reference genome, and, more importantly, we use the reference genome to provide enriched* input* for the de novo assembler. Furthermore, we assume that the reference is closely related to the target genome, and therefore the reference is directly used to determine order and orientation of the assembly contigs. In contrast, RACA focused on reliable order and orientation of the contigs. Amos, one of the most popular assisted assembly softwares, aligns reads to the reference genome and uses alignment and layout information to generate a new consensus sequence [9]. We note that the techniques presented in this paper are orthogonal to the ones used in the aforementioned works; that is, as future work, RECORD may be combined with other assisted assembly tools. In this paper, we focus on experimentally evaluating the power of the relatively simple techniques of our pipeline. We will show that, despite their simplicity, they may achieve surprisingly good results.

In the next section we describe our approach, a simple but surprisingly effective reference-assisted assembly technique, and the software that implements it. By design, this approach is most useful in cases when the reference and target genomes are closely related, and the coverage of the target genome by the experimental reads is relatively low such as multiplexing scenarios where multiple experimental DNA libraries are barcoded and pooled in a single sequencing lane. Subsequently we present the results of the experimental evaluation of RECORD and compare it to Amos [9], one of the most popular assisted assembly tools. We show that, under realistic conditions of approximately 1 percent divergence between reference genome and the studied sequence, our approach outperforms naive approaches and Amos (which excels in situations where the divergence is much higher). To ensure reproducibility and extensibility of our work, we evaluate our approach on several collections of publicly available next-generation sequencing data sets originating from various model organisms such as yeast (*S. pombe*), fruit fly (*D. melanogaster*), and plant (*A. thaliana*).

## 2. Implementation

**Figure 1:**
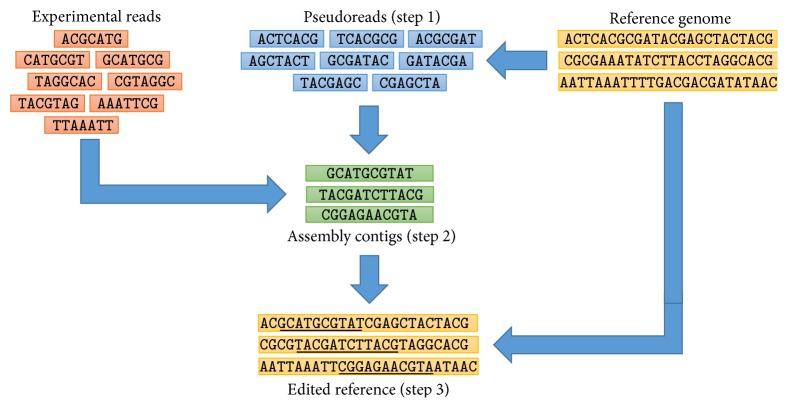
RECORD: Reference-Assisted Genome Assembly for Closely Related Genomes. The inputs of the pipeline, that is, the experimental reads and the reference genome, are illustrated in the top left and top right of the figure, respectively. Intermediate results produced in various steps of the analysis process are depicted. The dependency between these intermediate results is shown by arrows. In the illustration of the 3rd step, we underlined those segments of the edited reference which were replaced by one of the assembly contigs.

We propose RECORD, Reference-Assisted Genome Assembly for Closely Related Genomes. Our approach consists of the following steps (see Figure 1):

1. We generate pseudoreads from the reference genome. We generate pseudoreads in order to ensure that the coverage of the genome is large enough.
2. We obtain the contigs of the actual genome of the organism using a genome assembler, such as Velvet [17]. As input of the assembler, we propose to use the pseudoreads generated in the previous step together with the experimental reads.
3. We create an edited reference genome. The contigs obtained in the previous step may not cover the actual genome of the organism entirely, and, more importantly, the genome obtained in the previous steps may be fragmented into a relatively large amount of contigs. Therefore, the contigs obtained in the previous step will be mapped to the reference genome with MUMmer [18]. Using the reference genome and the mapped contigs, we produce a new genome, called edited reference, the segments of which are replaced according to the mapping. This step ensures that the edited reference is close to the true genome of the organism, while it covers as much regions of the genome as possible.

Below we give a detailed description of the above steps.

### 2.1. Generation of Pseudoreads from the Reference

**Figure 2:**
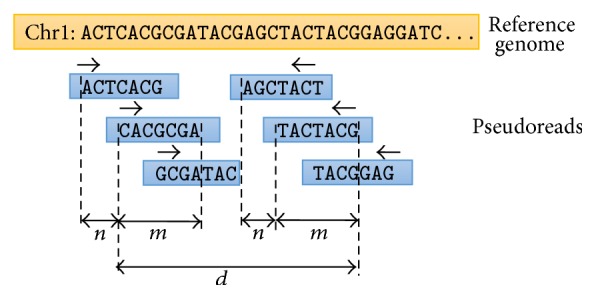
Generation of pseudoreads from the reference genome.

While generating pseudoreads from the reference, we make sure that these pseudoreads have uniform coverage and large enough overlaps so that they can “assist” the genome assembler, while it joins reads to contigs. In particular, we generate reads of length *m* from each chromosome beginning at positions 0, *n*, 2 · *n*,…, *k* · *n*,…, where *m* and *n* are parameters that can be set by the user. We generate paired-end reads; the first mate of the paired-end reads is generated directly from the reference, while the second mate is generated from its reverse complement, so that the resulting data has similar character as the paired-end reads in NGS experiments. The distance between the ends of the mates of the paired-end reads is *d*. This is illustrated in Figure 2.

Additionally, we associate each position of these pseudoreads with a relatively low quality score *q* in order to ensure that real reads have higher priority during the genome assembly process.

By default, whenever the opposite is not stated explicitly, we set *m* = 100, *n* = 30, *d* = 1000, and *q* = 10. The quality score is on the Phred scale from 0 to 93. We store the pseudoreads together with the quality scores as FastQ files [19] so that they can be used as input for the genome assembler Velvet.

### 2.2. Assisted Assembly

The second step of our approach leads to generation of assisted assembly contigs. To this aim, we combine pseudoreads generated in the previous step and experimental reads in one data set. Next, this data set is used as an input for a genome assembler. In principle, any assembler can be applied, but we use Velvet with its default parameters. However, the user may set values of the parameters according to his or her needs.

### 2.3. Editing the Reference

While editing the reference based on the alignment of the contigs produced by MUMmer, we have to take into account that contigs may be mapped ambiguously to the reference; that is, the same contig may be mapped to several segments of the reference. Moreover, the regions covered by different contigs may overlap and therefore some segments of the genome may be covered by several contigs. We resolve this ambiguity in two steps.

First, for each contig, we search for its* best* mapping to the reference. Conceptually, we can measure the quality of a mapping by the number of identical bases between the contig and the corresponding segment of the reference. This is estimated as

(1) $$\begin{array}{c} Q_{map}=L\times idy^{\left(ref\right)}, \end{array}$$

where *L* denotes the length of the mapped segment of the contig and idy^(ref)^ is the percentile identity between the contig and the corresponding reference segment as outputted by MUMmer. For each contig, out of its several mappings, we select the one that has the highest *Q*_map_ score.

Even though there is no theoretical guarantee that a particular contig corresponds to that segment of the genome to which it was mapped with highest *Q*_map_ score, we argue that, on one hand, the higher the identity is, the higher the likelihood that the mapping is correct is (i.e., the contig really originates from that segment of the genome to which it is mapped); on the other hand, the longer the mapped subsequence of the contig is, the higher the likelihood that the mapping is correct is. Therefore, the higher the above quality score is, the higher the likelihood of correct mapping is. Thus, we select for each contig the segment of the genome that has the highest *Q*_map_ score.

**Figure 3:**
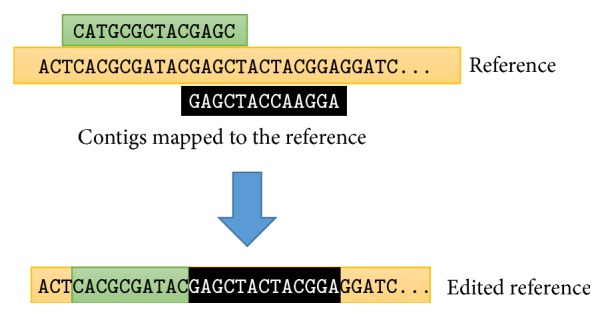
Resolution of ambiguity. First, for each contig, its best mapping is determined, and then the remaining ambiguity is resolved in greedy fashion by giving priority to the beginning of the contigs as shown in the figure.

As one can see in Figures 5 and 7, the ratio of ambiguously mapped contigs varies between 5% and 12% in most of our experiments. An exception is the case of* A. thaliana*, for which the proportion of ambiguously mapped contigs is between 30% and 40%. After selecting the best mapping for each contig, the remaining ambiguity may only arise from overlapping contigs as illustrated in Figure 3. According to our observations, selecting the best mapping for each contig greatly reduces the number of those genomic positions that are covered by multiple contigs. In particular, in both cases of* A. thaliana* and* D. melanogaster*, the selection of the best mapping of each contig reduced the number of multiply covered genomic positions by ≈90%. Furthermore, the overlapping segments of two contigs typically contain exactly the same or very similar genomic sequences. Therefore, the selection of the best mapping for each contig is able to eliminate vast majority of the ambiguity. In the light of these observations, Figure 3 shows an exceptional situation, in which two contigs overlap and the overlapping segments correspond to notably different genomic sequences. Despite the fact that such situations are exceptionally rare, in order to produce the edited reference, such ambiguity must be resolved. One possibility to resolve such ambiguity is to use the aforementioned quality scores and to prioritize the contig with higher *Q*_map_ score. In our prototypical implementation of the pipeline, we used an even simpler method: we resolved the ambiguity remaining after the selection of the best mapping in a greedy fashion by preferring the beginning of the contigs to the ends of the contigs as illustrated in Figure 3.

After resolving the ambiguity, the edited reference is produced by replacing the segments of the reference by the mapped contigs (or their segments).

### 2.4. Software

We implemented RECORD using Perl and Java programming languages. The main program is implemented in Perl programming language. The main program calls Velvet and the modules, for generation of pseudoreads and reference editing.

## 3. Results and Discussion

Our approach does not aim to reproduce the reference genome (which is used as input anyway), but we aim to recover the true genome of the organism which is unknown in case of real experiments. Consequently, the evaluation of any assembly software is inherently difficult. Therefore, in the following sections, we present evaluation on both simulated and real data. In case of simulated data, a gold standard is available, while the experiments on real data will show that our approach may be useful in real applications.

Next, we present the results of the experimental evaluation of our approach.

### 3.1. Baselines

In the experiments presented in the subsequent sections, we used two genome assemblers, Velvet [17] and Amos [9], as baselines. Velvet is a de novo genome assembler; that is, it assembles the genome directly from the experimental reads, whereas Amos is one of the most popular assisted genome assembly software tools; that is, Amos uses both the experimental reads and the reference genome of a genetically related organism in order to reconstruct the genome of the studied organism. Throughout the description of the experiments, with Velvet we refer to the case of using Velvet as standalone application, even though our approach, referred to as RECORD, uses by default Velvet as a component of the proposed pipeline.

We also tried to use further assisted genome assemblers, such as ARACHNE [11, 12] and IMR/DENOM [13]. While these softwares may excel in various general settings (such as using the reference genome of a species to reconstruct the genome of an other species), as far as we can judge, they do not seem to fit to our special setting of relatively* low coverage* (i.e., few experimental reads) and* very* closely related genomes. For example, in some cases, the outputted genome was the reference genome, which, on one hand, may be considered as reasonable if the actual genome and the reference genome are* highly* similar (i.e., they are* almost* the same); on the other hand, this is a trivial solution for the assisted assembly problem as the reference is one of the inputs of reference-assisted assembly methods.

### 3.2. Evaluation on Simulated Data

We simulate the scenario that the reference genome is given and we aim to reconstruct the actual genome of the studied organism, which we call* target genome*. In particular, we used the Evolver software tool [20] to generate the target genome. We used the genome from the example that comes with Evolver. This is an artificial mammalian genome of size of 30 megabases (Mb). The genome has three chromosomes. In order to allow for an unbiased evaluation, we produced the evolved genome following the example attached with Evolver. We used the original genome, that is, ancestral genome, as the reference genome, and we considered the evolved genome as the target genome. We generated one million paired-end short reads of length of 70 with wgsim [21] from the target genome. Subsequently, we tried to reconstruct the target genome from the generated paired-end reads and the reference genome both with our approach and two other state-of-the-art genome assemblers. Throughout the experiments on simulated data, we used Velvet with *k*-Mer size of *k* = 21. Finally, we compared the outputs of the assemblers with the target genome and quantitatively measured the quality of each of the assemblers according to the following criteria:

(1) TL, the total length of the assembly in Mb.
(2) N50; that is, we consider the set of largest contigs that together cover at least 50% of the assembly, and out of these contigs the length of the shortest one is denoted as N50.
(3) Error = 100% − IDY, where IDY is the percentile identity between the target genome and the genome reconstructed by the assembler. (Please note that IDY is different from idy^(ref)^. While idy^(ref)^ denotes the identity between an assembly contig and the corresponding segment of the* reference* genome, we use IDY to denote the identity between the output of the assembly and the* target* genome.) In order to calculate IDY, we map the genome reconstructed by the assembler to the target genome using the MUMmer software tool [18], and we calculated the weighted average of the percentile identities between the mapped segments and the target genome as outputted by MUMmer. In the weighted average, we used the length of the mapped segments as weights.
(4) Number of identical bases, which we calculated as IDY × TL.

Both in case of our approach and in case of the baselines, we evaluated both the contigs and the edited reference resulting from using the contigs. In case of evaluating edited reference for the baselines, we simply used the contigs outputted by the baselines in the third step of our approach and produced the edited reference.

Table 1 summarizes our results. The columns of the table show the total length (TL) of the assembly, N50, error, and the number of identical bases. As one can see, our approach, RECORD, is competitive with the other assemblers: considering the contigs produced by our pipeline, they have the highest N50 and the lowest error rates, while the edited reference produced by RECORD has the overall highest number of identical bases with the target genome.

**Figure 4:**
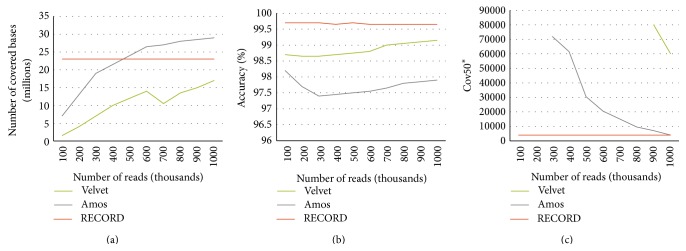
Comparison of the proposed approach (RECORD) with two state-of-the-art genome assemblers on data simulated with wgsim. In this experiment, we consider the evolved genome produced by Evolver as the target genome; the reference genome is the ancestral genome. The diagrams show the performance of the examined approaches according to various criteria as the function of the number of simulated reads that were used for the assembly. The diagram (a) shows the number of covered bases of the target genome; the diagram (b) shows the accuracy, that is, overall percentile identity between the assembly contigs and the corresponding segments of the target genome, while the diagram (c) shows the number of those largest contigs that together cover at least 50% of the target genome.

In a subsequent experiment, we varied the number of reads used for the assembly and evaluated the resulting contigs. These results are shown in Figure 4. The diagram (a) shows the number of bases in the target genome that are covered by the assembly contigs as function of the number of reads that were used. It is important to note that while Amos can provide overall better coverage of the sequence, it requires more reads (>500 k) for that. In the lower range of the number of reads available, it is outperformed by RECORD. It may be relevant for practical applications as the cost of the experiment usually depends on the number of reads produced. While in this simulated case the number of reads is relatively low for today NGS technology standards, it might be still relevant in multiplexing scenarios where multiple experimental DNA libraries are barcoded and pooled in a single sequencing lane.

The second diagram (b) shows the overall percentile identity between the target genome and the contigs of the assembly. The third diagram (c) shows the number of those largest contigs that together cover at least 50% of the target genome. As one can see, if only relatively few reads are available, our approach, RECORD, systematically outperforms the baselines by producing larger contigs, the most accurate and most complete assembly.

**Figure 5:**
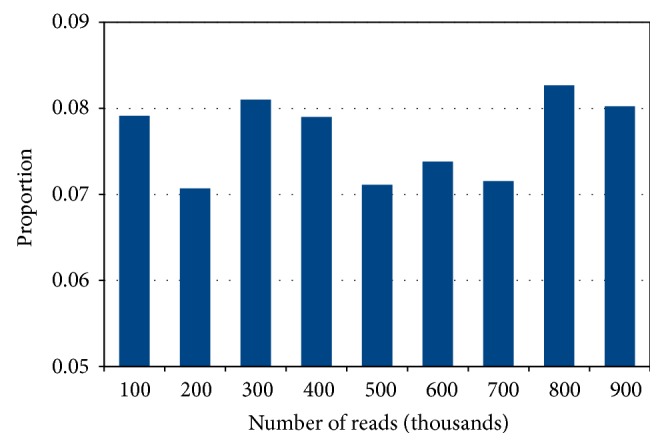
Proportion of ambiguously mapped contigs (before the selection of the best mapping for each contig) in case of various numbers of simulated reads.

In order to analyze our approach in more detail, we show in Figure 5 the proportion of ambiguously mapped contigs (before the selection of the best mapping for each contig). As one can see, the proportion of ambiguously mapped contigs varies between 7% and 8.5%.

We note that, from the point of view of applications, there is a substantial difference between the execution times of RECORD and Amos. For example, when using 300 thousand reads, producing the edited reference took approximately 1 hour for our approach, whereas it took 16 hours for Amos. We emphasize that this observation refers to the practical application of the software but not to the overall (theoretical) computational costs: much of the observed difference may be attributed to the fact that Velvet, which is used by default as assembler in the proposed pipeline, is able to run in parallel on multiple cores, whereas Amos can be used on one core at a time. Due to the fact that RECORD uses a de novo assembler as a component of the proposed pipeline, our approach is limited to middle-sized genomes that are closely related to the reference genome; therefore it is currently not applicable to the human and comparable genomes.

### 3.3. Evaluation on Real Data

The primary goal of the evaluation on real data was to show that our approach can be applied in real experiments.

#### 3.3.1. Assessment of the Accuracy in Comparison to the Baseline

As mentioned previously, in real-world settings, there is usually no gold standard available. Therefore, the assessment of the accuracy of the genome produced by any assembler is inherently difficult. For this reason, in the subsequent experiment, we evaluate the accuracy of the proposed method on real data indirectly. In particular, we assess the quality of the contigs and, more importantly, we compare our approach to the baselines in the following setting: we examine how well we can reconstruct the genome using relatively small subsets of all the available reads. These subsets are uniform random samples taken from the set of all the reads: each read has the same probability of being included in the sample. Paired-end reads are sampled together with their mates; that is, either both sequences corresponding to a particular paired-end read are selected or none of the sequences of that paired-end is selected.

In the aforementioned context, as gold standard, that is, target genome, we consider the genome produced by Amos when using* all* the reads for the assembly. We note that this leads to an evaluation in which Amos has an inherent advantage against our approach, as unfortunately we cannot have an unbiased reference.

We used real-world experimental reads graciously provided by dr Andrzej Dziembowski's group, coming from an unpublished ChIP-seq experiment in a yeast species. The data contained approximately 4.5 million paired-end short reads of length of 100.

**Figure 6:**
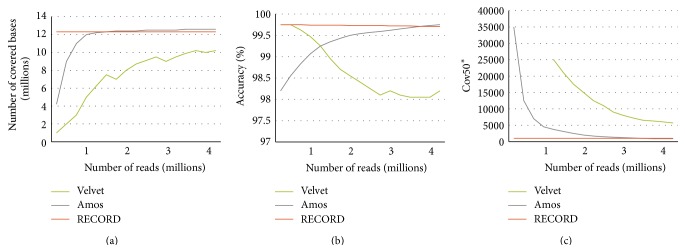
Comparison of the proposed approach (RECORD) with two state-of-the-art genome assemblers on real data. In this experiment, we compared assemblies resulting from various number of experimental reads to the assembly which is produced by Amos using all the experimental reads; that is, the target genome is the assembly produced by Amos using all the reads. In this case, the reference genome exhibits 99.7 percent identity with the result of Amos which is used as the gold standard. The diagrams follow the same structure as the one in Figure 4.

Figure 6 shows the results. The diagrams follow the same structures as the ones presented at the end of Section 3.2; that is, the diagram (a) shows the number of bases in the target genome that are covered by the assembly contigs as function of the number of reads that were used. The second diagram (b) shows the accuracy, that is, the overall percentile identity between the target genome and the contigs of the assembly. The third diagram (c) shows the number of those largest contigs that together cover at least 50% of the target genome. In all the three diagrams, the horizontal axis shows the size of the sample (i.e., the number of paired-end reads) used to assemble the genome. As one can see, our approach, RECORD, systematically outperforms the baselines in terms of accuracy and coverage of the genome. Note that, in case of using very few reads, Velvet achieves as good accuracy as our approach; however, the contigs it produces have very low coverage.

#### 3.3.2. Characteristics of the Assembly of Publicly Available Data Sets

In order to assist reproducibility of our results, we used publicly available real short read data from the NCBI Short Read Archive. We used data originating from three different species: plant (*A. thaliana*), fly (*D. melanogaster*), and yeast (*S. pombe*). The identifiers of the short read collections are shown in the third column of Tables 2 and 3.

We set the *k*-mer size for the assembly, that is, the second step of our approach, in accordance with length of the short reads in the archive and the (approximate) size of the target genome. In particular, similarly to the previous experiments, we set *k* = 21 for yeast (short read length = 44), while we used slightly larger settings for the other two species: we set *k* = 45 in case of flower (short read length = 80) and *k* = 25 for fly (short read length = 36).

Tables 2 and 3 show the most important characteristics of the resulting assembly contigs and the edited reference. In particular, the fourth column of Table 2 shows the total length of the assembly contigs; the fifth column shows the number of all the contigs, while in the sixth column the N50 of the contigs is shown. The last column shows the coverage of experimental reads calculated as follows:

(2) $$\begin{array}{c} \text{Cov}=\frac{\text{read length}\times \text{number of reads}}{\text{genome size}}. \end{array}$$

The third column of Table 3 shows the total length of the replaced segments, while the fourth and fifth columns show in percent the ratio of the length of the replaced segments relative to the length of the reference and the total length of the assembly contigs, denoted as % ref and % asm, respectively. The sixth column of Table 3 shows the number of contigs that were used while editing the reference. The last column of Table 3 shows the overall percentile identity between the edited reference and the original reference.

**Figure 7:**
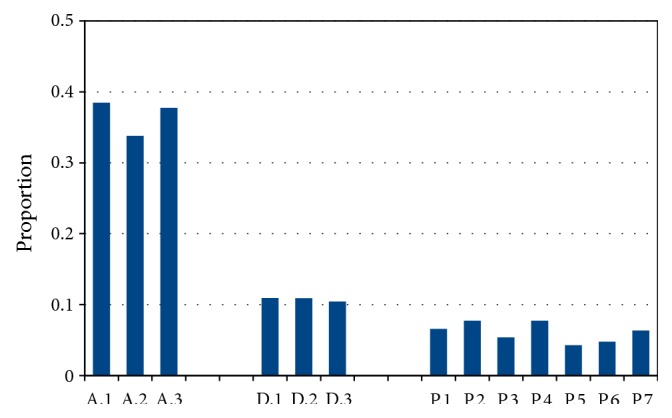
Proportion of ambiguously mapped contigs (before the selection of the best mapping for each contig) in case of experiments on publicly available data sets.

Figure 7 shows the proportion of ambiguously mapped contigs (before the selection of the best mapping for each contig) for each experiment shown in this section. As one can see, the proportion of ambiguously mapped contigs varies between 4% and 8% in case of* S. pombe*; it is around 10% in case of* D. melanogaster*; and it is remarkably higher, around 35%, for* A. thaliana*.

The results in Table 3 show that the total length of the assembly is close to the genome size, indicating the completeness of the assembly. However, the relatively large number of contigs in the raw assembly output can be seen as an indication that the assembler had difficulties with particular regions of the genome, and therefore a large number of short fragments may have been produced. This is especially visible in the case of* D. melanogaster*, where two factors influencing the quality of assembly are combined: low read coverage and low read length.

According to the proposed procedure of editing the reference, a contig may be left out if it can not be mapped to the reference or if MUMmer considers it too short to produce a useful alignment. As we can see, the number of contigs contributing to the edited reference is substantially less than the total number of contigs. However, in terms of length, almost the entire assembly is used; for example, in each of the* D. melanogaster* data sets the edited assembly utilizes ~11% of all contigs, covering over 95% of the assisted assembly. This shows that reference editing relies on a moderate amount of long contigs rather than on a bulk of short ones.

The edited part of the genome in* A. thaliana* is slightly smaller than in* D. melanogaster*, but the number of contributing contigs is slightly larger. Therefore, contigs obtained for the former organism are generally shorter than those obtained for the latter (this is also in accordance with the observation that N50 of* A. thaliana* is ~3× lower than N50 of* D. melanogaster*). Shorter contigs are more likely to be nonuniquely mapped, as we can observe on Figure 7; the proportion of ambiguously mapped contigs is similar to the proportion obtained in simulated data for* S. pombe* and* D. melanogaster*, while it is remarkably higher for* A. thaliana*.

Percentages of replaced segments in genome editing (% ref and % asm) are also similar to those observed in simulated data for two of our species (*A. thaliana* and* S. pombe*), while they are slightly lower for* D. melanogaster*. This behavior is explained by the difference in the coverage, which is in* D. melanogaster* an order of magnitude lower than in the two other species. The results indicate that the outputted genomes are closely related to the reference. This is expected, since the genomes of individuals are close to a reference genome of the respective species.

Overall, the results on real-world data are similar to those on simulated data (in some respects, e.g., N50, even better). Visibly more variability is observed between results on real data sets with different characteristics: read length, coverage, and so forth.

## 4. Conclusions

In this paper, we proposed a new approach for reference-assisted assembly of closely related genomes. Our approach takes into account that the actual genome of the studied organism may be slightly different from the reference genome of that species leading to potentially fewer errors in downstream analyses of the sequenced read abundances.

We have assessed the performance of our method on an artificially simulated mutated eukaryotic genome, showing that RECORD produces contigs with very low error rate (less than 0.5 percent) and after merging them with the original assembly leading to error rates smaller than in simpler de novo assembly technique (Velvet) as well as more general assisted assembly approach (Amos).

Further examination of the results in comparison to Amos and simple Velvet indicated that our approach is most useful in the case where we have relatively few reads at our disposal; both of the competing tools struggled with the data sets where the number of reads was low.

The same seems to be true in case of a real data set that we analyzed in Section 3.3.1. Even though the numbers of reads are much higher, we can still see the difference between our method and the more traditional approaches. Even though the genome size is small, we can see that RECORD shows clearly superior accuracy with up to 3 million reads and all measures are clearly better at approximately 1 million reads.

Finally, we apply RECORD to more than 10 publicly available data sets from Short NCBI Read Archive to show its applicability in practical situations. We can see in all cases that not only is RECORD able to produce results for much larger genomes (up to 140 Mb) but the estimated divergence between the examined genome and the reference is close to one percent where we can expect RECORD to perform better than its examined alternatives.

We provide a prototype implementation of this approach as a set of scripts. It is available for download at our supplementary website together with most of the data published in the study allowing the readers to replicate our results and adapt the method for specific applications.

## Availability and Requirements

 Project name: RECORD Genome Assembler
 Project home page: http://sourceforge.net/projects/record-genome-assembler/
 Operating system(s): Linux
 Programming language: Perl, Java
 Other requirements: Velvet, MUMmer
 License: Open Source
 Any restrictions to use by nonacademics: no.

## Figures and Tables

**Table 1:** Evaluation on simulated data.

Assembly	TL (Mb)	N50	Error (in %)	Id. Bases (Mb)
Contigs				
Velvet	18.20	213 b	0.85	18.05
Amos	28.82	1834 b	2.09	28.22
RECORD	25.81	2055 b	0.41	25.70
Edited reference				
Velvet	30.00	10 Mb	1.39	29.58
Amos	30.00	10 Mb	1.03	29.69
RECORD	30.00	10 Mb	0.59	29.82

**Table 2:** Assembly of real experimental reads (contigs).

Species	Number	Experimental reads	Length (Mb)	# ctgs	N50	Cov
*A. thaliana *	A.1	[SRR402840, SRR402839]	113.2	250148	19464	39.6x
	A.2	[SRR402842, SRR402841]	112.4	296438	18916	24.4x
	A.3	[SRR402844, SRR402843]	113.1	243375	20494	30.5x

* D. melanogaster *	D.1	[SRR066834, SRR066831]	122.6	460842	58648	2.6x
	D.2	[SRR066835, SRR066832]	122.6	461044	58535	2.1x
	D.3	[SRR066836, SRR066833]	122.6	460200	58415	2.4x

* S. pombe *	P.1	[SRR948260, SRR948250]	15.2	10384	8934	32.3x
	P.2	[SRR948261, SRR948251]	12.2	8129	11132	18.3x
	P.3	[SRR948262, SRR948252]	12.1	8571	6696	26.5x
	P.4	[SRR948266, SRR948272]	12.1	7589	10144	21.5x
	P.5	[SRR948267, SRR948273]	12.0	9214	4927	31.6x
	P.6	[SRR948268, SRR948274]	12.0	8964	5696	28.1x
	P.7	[SRR948269, SRR948275]	12.1	8269	8918	27.4x

**Table 3:** Assembly of real experimental reads (edited reference).

Species	Number	ed.len. (Mb)	% ref	% asm	# ctgs	% IDY
*A. thaliana *	A.1	109.9	91.8	97.3	51769	99.967
	A.2	106.4	89.0	95.5	58052	99.918
	A.3	109.8	91.8	97.3	54185	99.972

*D. melanogaster *	D.1	117.4	82.1	95.8	50598	99.985
	D.2	117.0	81.8	95.4	50606	99.986
	D.3	117.2	82.0	95.6	50630	99.986

*S. pombe *	P.1	12.0	95.1	78.9	3548	99.995
	P.2	12.0	95.2	98.4	2931	99.994
	P.3	12.0	95.0	99.2	4249	99.996
	P.4	12.0	95.3	99.2	3054	99.994
	P.5	11.9	94.6	99.2	5287	99.996
	P.6	12.0	94.9	100.0	4762	99.996
	P.7	12.0	95.3	99.2	3465	99.994
